# Papillary Thyroid Carcinoma in a Patient With a Germline Titin (TTN) Mutation

**DOI:** 10.7759/cureus.104053

**Published:** 2026-02-22

**Authors:** Summiya Nasim, Kamran Mushtaq, Emily Schroeder

**Affiliations:** 1 Internal Medicine, Parkview Health, Fort Wayne, USA; 2 Internal Medicine, Northeast Internal Medicine Associates, LaGrange, USA; 3 Endocrinology, Parkview Health, Fort Wayne, USA

**Keywords:** amiodarone-induced thyrotoxicosis, dilated cardiomyopathy, papillary thyroid microcarcinoma, thyroid cancer survelliance, ttn germline mutation

## Abstract

The titin (TTN) gene encodes the largest known human protein and is essential in maintaining cardiac sarcomere structure. Germline TTN mutations are strongly linked to dilated cardiomyopathy and arrhythmias, and emerging evidence suggests a possible relationship between TTN alterations and oncologic processes. Owing to the large size of the TTN gene, recurrent mutations are frequently observed across cancer datasets, but the clinical and biological significance of these alterations, particularly in the germline setting, remains incompletely understood. This case presents a patient with a germline TTN mutation who was diagnosed with papillary thyroid carcinoma after he developed amiodarone-induced thyrotoxicosis.

## Introduction

Titin (TTN) is the largest known human protein and plays a critical structural and biomechanical role within the cardiac sarcomere. Pathogenic variants in TTN, particularly truncating mutations, are among the most common genetic causes of dilated cardiomyopathy and represent a major contributor to inherited cardiomyopathies [1].

With the increasing availability of large-scale genomic sequencing, TTN mutations have been identified not only in cardiac disease but also across a wide spectrum of malignancies. Because TTN is one of the largest genes in the human genome, it is frequently mutated in cancer sequencing datasets, often reflecting background somatic variation rather than a direct oncogenic driver [2]. Large pan-cancer analyses have demonstrated that TTN mutations correlate with overall tumor mutational burden and may serve as surrogate markers of genomic instability [3].

In thyroid cancer specifically, TTN mutations have been reported in genomic studies and have been associated with higher tumor mutational burden and adverse prognostic features, although their precise role in thyroid tumorigenesis remains incompletely understood [4]. Differentiated thyroid carcinoma is a common endocrine malignancy characterized by diverse genetic and molecular alterations that influence disease behavior and clinical outcomes [5].

Most genetic alterations identified in differentiated thyroid cancer are somatic; however, the clinical significance of germline variants in cancer-associated genes such as TTN remains an evolving area of investigation [5,6]. Understanding whether germline TTN mutations contribute directly to oncogenesis or instead modify disease susceptibility and tumor behavior has important implications for cancer screening, prognosis, and personalized management strategies [6].

We report a patient with a known germline TTN mutation who presented with hyperthyroidism and was ultimately diagnosed with an incidentally discovered papillary thyroid microcarcinoma. While papillary thyroid microcarcinoma is common in the general population and frequently identified incidentally, this case represents a coexistence of findings rather than evidence of a causal relationship between germline TTN mutation and thyroid carcinogenesis. This case highlights the diagnostic complexity of evaluating thyroid dysfunction in genetically predisposed individuals and underscores the importance of distinguishing primary thyroid disease from medication-related etiologies, including amiodarone-induced thyrotoxicosis [7]. Furthermore, this report contributes to the growing literature exploring potential associations between germline TTN mutations, thyroid dysfunction, and malignancy.

## Case presentation

A 60-year-old male patient with a history of a germline TTN mutation, atrial fibrillation, and recurrent ventricular tachycardia requiring Implantable Cardioverter-Defibrillator (ICD) placement presented with symptoms of new-onset hyperthyroidism. He had been treated with amiodarone for more than two years, which was discontinued approximately six months prior to presentation. He reported palpitations, fatigue, and heat intolerance. Initial laboratory evaluation demonstrated suppressed thyroid-stimulating hormone (TSH) with elevated free thyroxine (T4) and free triiodothyronine (T3) levels, consistent with thyrotoxicosis (Table 1).

**Table 1:** Initial laboratory findings Laboratory results are shown with corresponding institutional reference ranges at the time of initial presentation.

Laboratory test	Patient value	Reference range
Thyroid-stimulating hormone (TSH; µIU/mL)	<0.01	0.25–4.50
Free thyroxine (free T4; ng/dL)	3.45	0.80–1.80
Free triiodothyronine (free T3; pg/mL)	10.30	2.30–3.80
Thyroid-stimulating immunoglobulin (TSI; %)	<89	<140
Thyroid peroxidase antibody (TPO Ab; IU/mL)	<9	0–31

**Figure 1:**
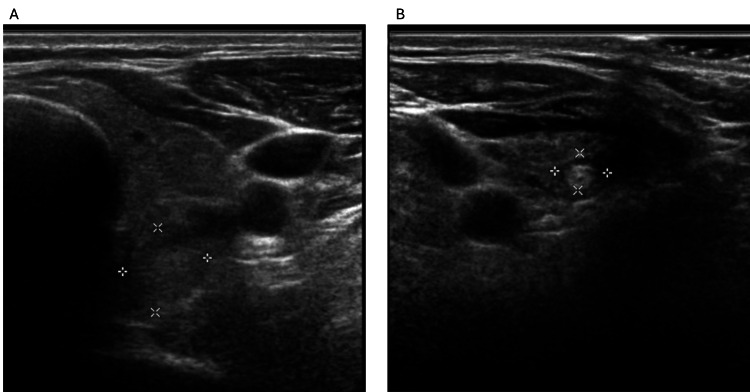
Thyroid ultrasound (transverse views) (A) Transverse view of a 1.13 × 1.11 cm left-lobe thyroid nodule that underwent fine-needle aspiration (FNA) biopsy (Bethesda IV) with Afirma genomic sequencing classifier “suspicious,” later confirmed benign at surgery; (B) Transverse view of a 0.68 × 0.48 cm right-lobe thyroid nodule that was not initially biopsied but was subsequently resected and proven to be papillary thyroid microcarcinoma.

The thyroid ultrasound revealed a multinodular goiter with multiple cystic lesions and a 5 mm solid nodule in the right lower lobe. The patient was started on methimazole with clinical improvement. A follow-up ultrasound one year later demonstrated a newly developed 11 × 11 × 15 mm mixed-echogenic solid nodule in the left lower lobe (Figure 1), while the right-sided nodule remained stable.

Fine-needle aspiration (FNA) cytology was Bethesda category IV [8], suspicious for follicular neoplasm, and Afirma genomic sequencing classifier (GCS; Veracyte, Inc., California, USA) [9], indicating an approximate 50% risk of malignancy.

Total thyroidectomy was performed based on the combination of Bethesda IV cytology and a suspicious Afirma GSC result, in accordance with standard surgical management for indeterminate nodules with molecular risk stratification. Additional considerations included the patient’s symptomatic thyrotoxicosis, underlying cardiac comorbidities, and the need for definitive management to avoid recurrent thyroid dysfunction. Surgical pathology revealed a 1.5 mm papillary thyroid microcarcinoma in the right lower lobe without evidence of lymphovascular invasion. Postoperative laboratory findings are summarized in Table 2.

**Table 2:** Postoperative laboratory findings Postoperative laboratory findings obtained following total thyroidectomy demonstrate normalization of thyroid function parameters. Reference ranges correspond to institutional standards.

Laboratory test	Patient value	Reference range
Thyroid-stimulating hormone (TSH; µIU/mL)	1.19	0.25–4.50
Free thyroxine (Free T4; ng/dL)	1.79	0.80–1.80
Thyroglobulin (ng/mL)	0.2	2.8–40.9
Thyroglobulin antibody (IU/mL)	11	0–60

The patient was initiated on levothyroxine replacement therapy and has demonstrated stable thyroid hormone levels with no evidence of recurrence.

## Discussion

TTN is the largest known human protein and plays a fundamental structural and biomechanical role within the cardiac sarcomere. Truncating variants in the TTN gene are among the most common genetic causes of dilated cardiomyopathy and are well-established contributors to inherited and acquired cardiac disease [1]. Beyond cardiology, increasing use of large-scale genomic sequencing has revealed TTN alterations across multiple malignancies. In thyroid cancer specifically, TTN mutations have been reported within tumor sequencing datasets and have been associated with adverse clinical outcomes in some studies, although their biological significance remains uncertain [2].

Because TTN is one of the largest genes in the human genome, it is frequently mutated across cancer sequencing studies, often reflecting background somatic variation rather than a direct oncogenic driver effect. Oh et al. demonstrated that TTN mutation burden may serve as a surrogate marker for overall tumor mutational burden across diverse cancers [3]. Similarly, pan-cancer analyses of mutational signatures have shown that recurrent mutations in large genes such as TTN are commonly observed without necessarily conferring functional oncogenic consequences [4].

In this case, the patient developed hyperthyroidism in the context of prior amiodarone exposure, a well-recognized cause of thyrotoxicosis due to iodine excess and thyroid hormone dysregulation. Amiodarone-induced thyrotoxicosis may occur through iodine-mediated increased hormone synthesis (type 1) or destructive thyroiditis (type 2), both of which can produce inflammatory changes within thyroid tissue. Although chronic inflammatory processes theoretically could alter thyroid architecture or contribute to nodule detection during evaluation, current evidence does not support a direct role for amiodarone in thyroid carcinogenesis. In this patient, amiodarone exposure is therefore best interpreted as a contributor to thyroid dysfunction and diagnostic evaluation rather than a causative factor in the development of papillary thyroid microcarcinoma [5].

Differentiated thyroid cancers, including papillary thyroid carcinoma, are characterized by diverse molecular alterations affecting key signaling pathways involved in cell proliferation, differentiation, and survival [6]. Common driver mutations such as B-Raf proto-oncogene, serine/threonine kinase (BRAF), Rat Sarcoma viral oncogene homolog family (RAS), and Rearranged during Transfection/Papillary Thyroid Carcinoma (RET/PTC) rearrangements are well established in thyroid tumorigenesis, whereas the role of large structural genes such as TTN remains incompletely understood [7].

The diagnostic evaluation of indeterminate thyroid nodules has evolved with the incorporation of molecular testing platforms such as the Afirma GSC, which aim to improve malignancy risk stratification and guide surgical decision-making. While molecular classifiers enhance diagnostic accuracy, results must be interpreted in conjunction with clinical, radiologic, and cytopathologic findings [10].

Large-scale cancer sequencing efforts have further emphasized that recurrent mutations in large genes, including TTN, are frequently detected across tumor types and may reflect underlying genomic instability rather than lineage-specific oncogenesis [11]. As molecular characterization of thyroid cancer continues to advance, understanding how germline genetic variants may influence cancer susceptibility, tumor behavior, or response to therapy remains an important area of investigation [12].

The coexistence of a germline TTN mutation and papillary thyroid microcarcinoma in this patient highlights the diagnostic complexity encountered in genetically predisposed individuals. Although causality cannot be established from a single case, this report underscores the importance of cautious interpretation of genomic findings and supports continued research into the potential clinical implications of germline TTN variants in endocrine malignancies.

## Conclusions

This case highlights the coexistence of a germline TTN mutation and an incidentally discovered 1.5 mm papillary thyroid microcarcinoma. Given the small size of the tumor and its frequent incidental occurrence in the general population, the malignancy is best interpreted as an incidental finding rather than a clinically aggressive or causally linked event. Nevertheless, the case underscores the diagnostic challenges of evaluating thyroid abnormalities in patients with underlying genetic cardiomyopathies and complex medical histories. Although a causal relationship between germline TTN variants and thyroid carcinogenesis cannot be established, this report raises awareness of the potential interplay between inherited genomic susceptibility and malignancy. Careful longitudinal surveillance and multidisciplinary evaluation are essential in such patients. Further studies are needed to clarify whether germline TTN mutations influence cancer risk, tumor behavior, or clinical outcomes in endocrine malignancies.
